# Two-stage laparoscopic surgery for incarcerated umbilical Littre’s hernia in severely obese patient: a case report

**DOI:** 10.1186/s40792-020-01008-3

**Published:** 2020-10-01

**Authors:** Yu Ariyoshi, Takayuki Suto, Akira Umemura, Hisataka Fujiwara, Shingo Yanari, Noriyuki Uesugi, Tamotsu Sugai, Akira Sasaki

**Affiliations:** 1 Department of Surgery, Morioka Municipal Hospital, 5-15-1 Motomiya, Morioka, Iwate 020-0866 Japan; 2 Department of Surgery, Iwate Medical University School of Medicine, 2-1-1 Idaidori, Yahaba-cho, Shiwa-gun, Iwate, 028-3694 Japan; 3 Department of Molecular Diagnostic Pathology, Iwate Medical University School of Medicine, 2-1-1 Idaidori, Yahaba-cho, Shiwa-gun, Iwate, 028-3694 Japan

**Keywords:** Meckel’s diverticulum, Umbilical hernia, Littre’s hernia, Laparoscopic hernia repair, Intraperitoneal onlay mesh (IPOM)

## Abstract

**Background:**

Littre's hernia containing Meckel's diverticulum is an extremely rare disease. We report an adult case of two-stage laparoscopic surgery for incarceration of Meckel's diverticulum in an umbilical hernia.

**Case presentation:**

The case involved a 23-year-old, severely obese man with BMI 36.5 kg/m^2^. After experiencing effusion from the umbilicus for 2 months, and was referred from a local dermatologist. We diagnosed an infected urachal remnant, and antibiotic therapy was performed first. Surgery was planned for after the infection disappeared. During follow-up, effusion from the umbilicus took on an intestinal fluid-like character, so we diagnosed small intestinal cutaneous fistula and performed surgery. Under laparoscopy, we found a Meckel's diverticulum incarcerated in an umbilical hernia. The diverticulum was resected first, and the incarceration was released. The umbilicus was infected, so we planned repair of the umbilical hernia in a second surgery. The postoperative course was uneventful and the patient was discharged on postoperative day 5. One month after the initial operation, we confirmed that there were no signs of infection, and performed umbilical hernia repair using the laparoscopic intraperitoneal onlay mesh (IPOM) repair. Postoperative progress was uneventful and he was discharged on postoperative day 4. No recurrence or infection was observed until 8 months postoperatively.

**Conclusions:**

We performed dissection of the diverticulum and umbilical hernia repair for the incarcerated umbilical Littre's hernia under laparoscopy in a severely obese patient. The risk of mesh infection seems to be avoidable using a two-stage surgery, and the risk of recurrence can be reduced using the IPOM repair compared with simple suture closure.

## Background

The recurrence rate of umbilical hernia has recently been reported as significantly lower following intraperitoneal onlay mesh (IPOM) repair than after simple suture closure [1, 2]. The laparoscopic IPOM repair is attracting increasing attention because it allows observation of the hernia from within the abdominal cavity, so that the hernial orifice can be identified accurately, and thus covered with a mesh of sufficient margins.

Hernia containing Meckel's diverticulum is called Littre's hernia. Adult Littre’s hernia is rare and a systematic review from 1954 to 2018 confirmed only 53 cases [3]. In that period, femoral hernia was most frequently reported (21 cases, 39.6%), followed by inguinal hernia (18 cases, 34.0%). Umbilical hernia was very rare, with only 6 cases (11.3%).

We report a case in which a two-stage laparoscopic surgery was used for incarcerated umbilical Meckel's diverticulum with infection. First, we released the incarcerated Littre’s hernia and dissected out the Meckel's diverticulum, and second, we performed laparoscopic IPOM repair. Here, we report details of the case together with a discussion of the literature.

## Case presentation

**Fig. 1:**
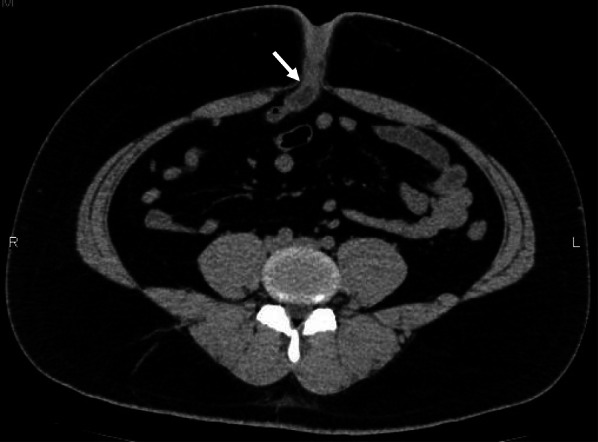
CT image at first visit. A luminal structure with liquid retention is evident just below the umbilicus (arrow)

**Fig. 2:**
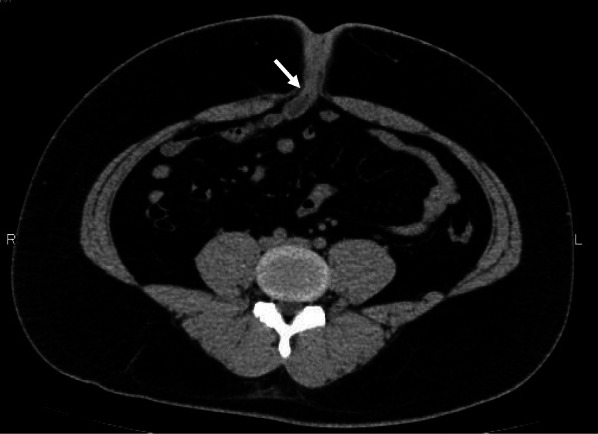
CT image before surgery. Findings resemble those at the first visit, showing a luminal structure with liquid retention just below the umbilicus (arrow)

The patient was a 23-year-old man with no past medical history. After experiencing effusion from the umbilicus and redness of it for 2 months, he presented to a local dermatologist. He was repeating relief and worsening by antibiotic administration. He was referred local surgery clinic, but it was not getting better. He was referred to our hospital without improvement after application of gentamicin sulfate ointment and oral administration of cefcapene pivoxil hydrochloride hydrate. Examination on presentation measured the patient’s height at 173.7 cm, weight at 110 kg, and BMI at 36.5 kg/m^2^; he was thus classified as severely obese. Redness was observed around the umbilicus, with expression of exudate on compression. No general symptoms such as fever, abdominal pain, or vomiting were observed at the time of examination or during the subsequent course. Blood testing showed a C-reactive protein level of 0.25 mg/dL and a white blood cell count of 8400/μL, showing no increase in inflammatory reaction. No other unusual findings were noted. On the first visit, computed tomography (CT) showed a luminal structure with fluid retention just below the umbilicus, but no intestinal dilation (Fig. 1). Based on this, we suspected infection of a urachal remnant and planned to perform surgery after the infection had been resolved with administration of oral levofloxacin hydrate tablets and topical gentamicin sulfate ointment. However, during follow-up, drainage from the umbilicus changed to resemble intestinal fluid, and the extent of the skin inflammation widened. We thus diagnosed small intestinal cutaneous fistula and performed surgery. When further CT was performed preoperatively, the findings resembled those at the first visit (Fig. 2).

**Fig. 3:**
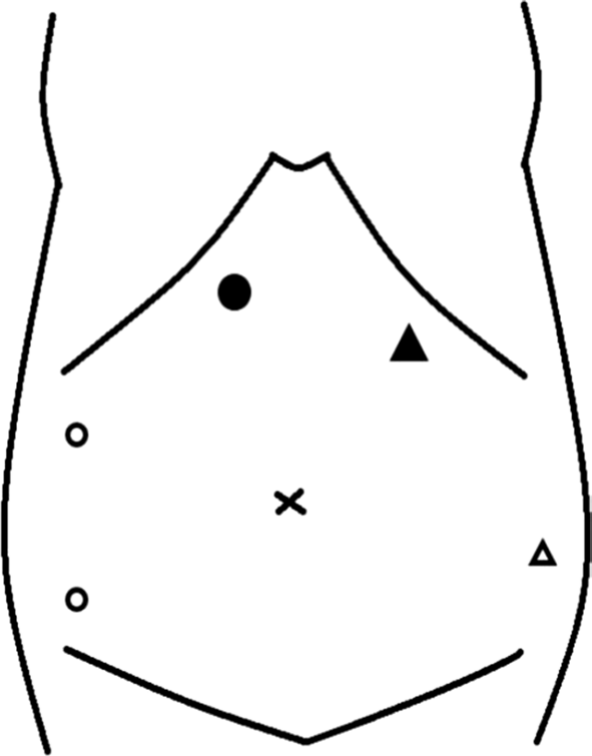
Trocar placement in first and second operations. ● A 12-mm trocar in the first operation. 〇 A 5-mm trocar in the first operation. ▲ A 12-mm trocar in the second operation. △ A 5-mm trocar in the second operation

**Fig. 4:**
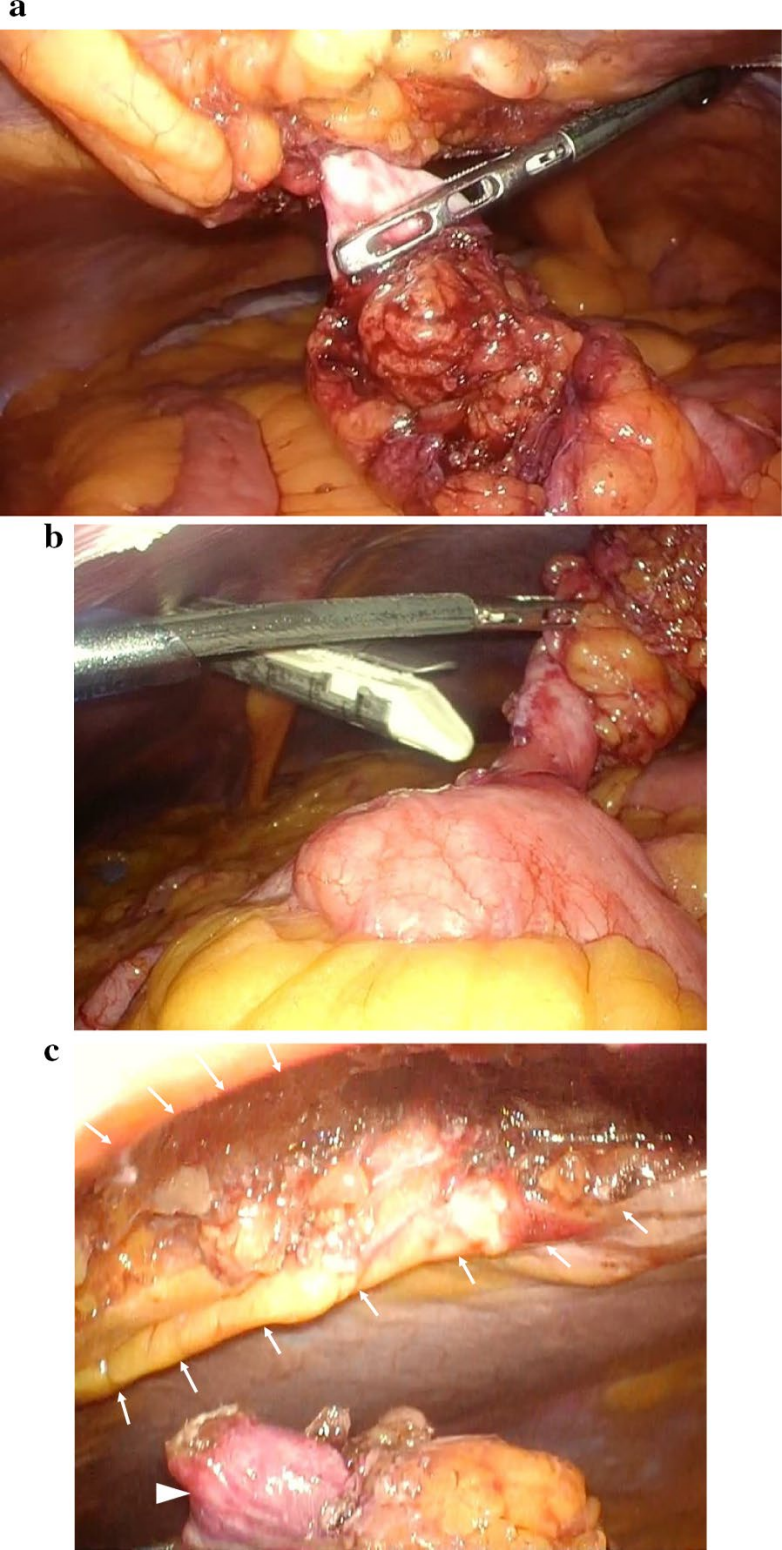
Intraoperative photograph from the first surgery (relief of incarceration and diverticulectomy). **a** Meckel’s diverticulum in the umbilical hernia. **b** Dissection of Meckel's diverticulum using a linear stapler. **c** Hernial orifice (arrows) and Meckel's diverticulum (arrowhead) after relieving incarceration

**Fig. 5:**
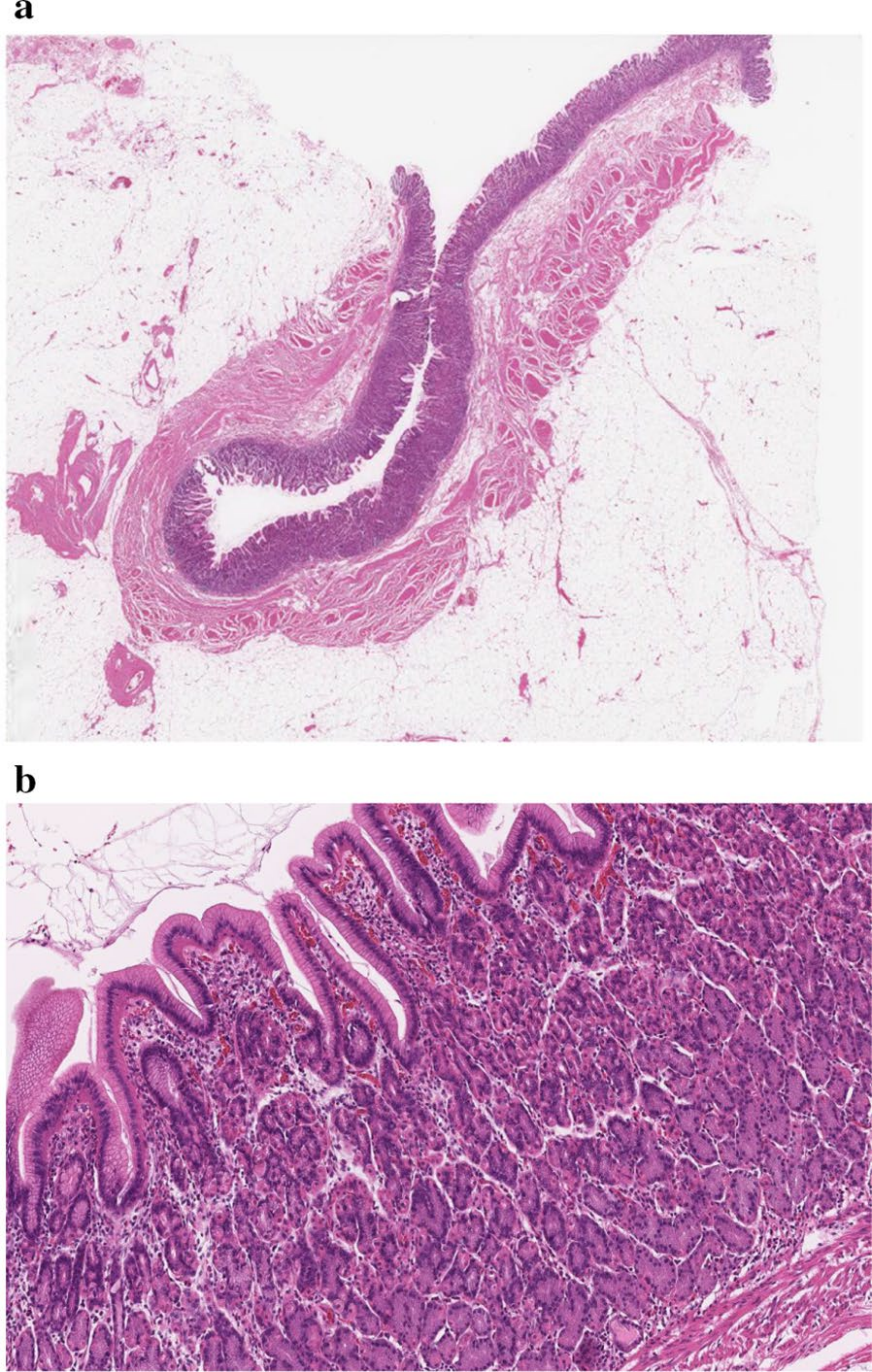
Pathological findings. **a** True diverticulum with a muscular layer is evident. **b** Presence of ectopic gastric mucosa is confirmed

For the operation, a 12-mm trocar was placed in the right epigastric region using the open method. Pneumoperitoneum was performed at 10 mmHg, and two 5-mm trocars were placed in the right lumbar region under observation with a 10-mm forward-viewing laparoscope (Olympus, Tokyo, Japan) (Fig. 3). When the inside of the abdominal cavity was observed, part of the intestinal wall showed Richter-type incarceration in the umbilicus. Observation after peeling diagnosed a hernia with incarcerated Meckel's diverticulum (Fig. 4a). An ultrasonic coagulation cutting device (SonoSurg®; Olympus) was used to remove adhesions and adipose tissue from around the hernia, exposing the intestinal wall and hernia orifice, but release of the incarceration by pulling proved difficult. We therefore decided to dissect the Meckel's diverticulum first. A 5-mm deflectable-tip videoscope (ENDOEYE FLEX™; Olympus) was inserted from the 5-mm trocar on the caudal side of the right lumbar region and a linear stapler (Echelon®; Ethicon, Cincinnati, OH, USA) was inserted from the 12-mm trocar to divide the diverticulum in the longitudinal direction of the small intestine (Fig. 4b). The hernial orifice (about 3 cm) at the incarcerated part of the Meckel's diverticulum was incised with an ultrasonic coagulation cutting device, and the separated Meckel's diverticulum was returned to the abdominal cavity from the hernia (Fig. 4c). The Meckel's diverticulum was then excised using a retrieval bag (B. Braun, Melsungen, Germany) from the 12-mm trocar under confirmation using a 5-mm deflectable-tip videoscope. The umbilicus was a cutaneous fistula accompanied by infection, so we did not perform one-stage hernia repair because of the high risk of mesh infection. Thus assuming a two-stage laparoscopic repair, Seprafilm (Sanofi, Paris, France) was applied to the hernial orifice and abdominal cavity to prevent adhesions. The 12-mm trocar wound was sutured with 0 Monosyn® (B. Braun) using EndoClose™ (Medtronic, Minneapolis, MN, USA), and all wounds were closed by dermal suturing with 4-0 Biosyn™ (Medtronic). Lidocaine hydrochloride (1%) was infiltrated into the port sites at the end of surgery. The operating time was 94 min, and intraoperative hemorrhage was 3 mL. Histological examination revealed that the diverticulum was true with a muscle layer (Fig. 5a) and included mucosa of the fundic gland and crypt epithelium. The diagnosis was therefore Meckel's diverticulum with ectopic gastric mucosa (Fig. 5b).　Incarcerated Meckel’s diverticulum had some fibrosis.

Immediately postoperatively, the exudate flow from the umbilicus disappeared, and inflammation around the umbilicus was improved. The postoperative course was uneventful, and oral intake was resumed on postoperative day 2. Flomoxef sodium was administered at 2 g/day for 2 days postoperatively, and the patient was discharged on postoperative day 5.

**Fig. 6:**
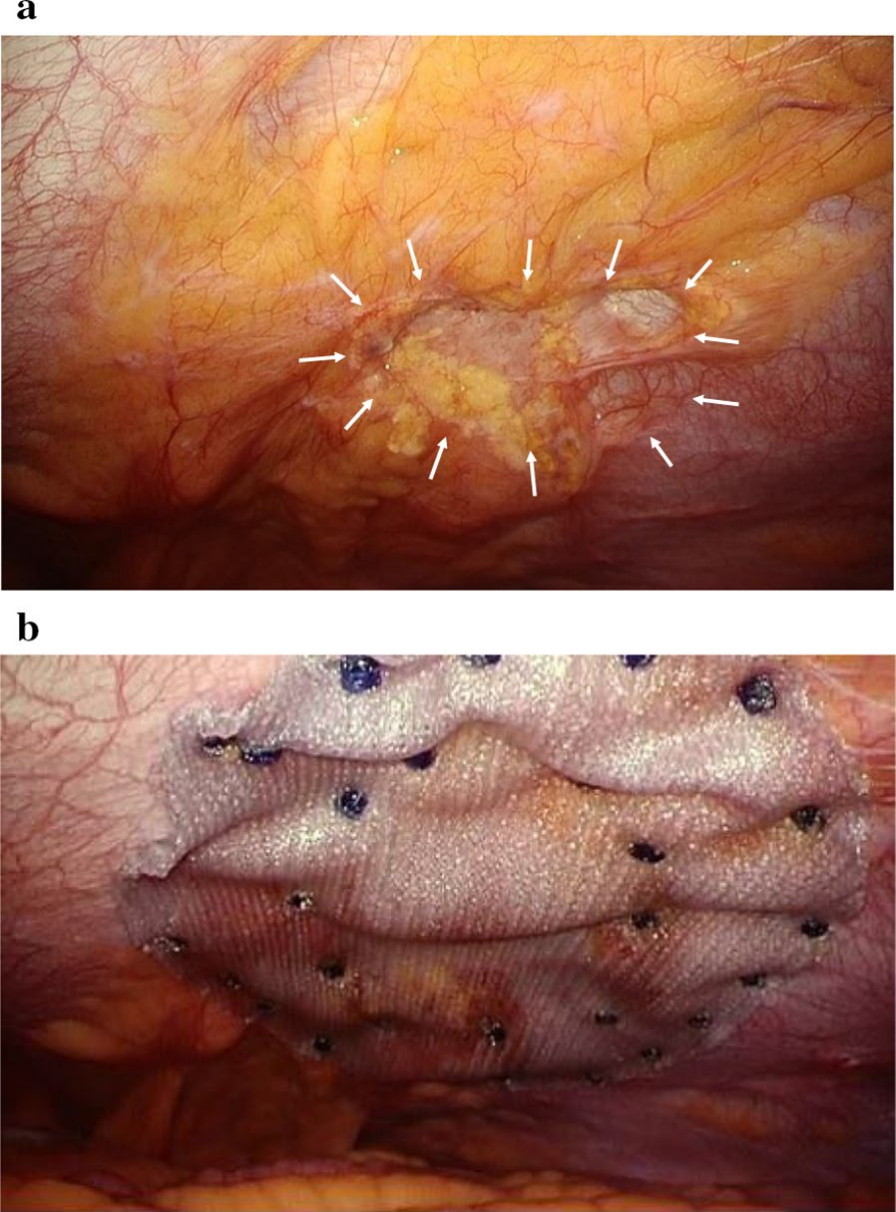
Intraoperative photograph from the second (hernial repair) surgery. **a** Hernial orifice shows scarring (arrows). **b** Mesh is secured using the double-crown method

One month postoperatively, we confirmed the absence of any signs of umbilical infection on follow-up at the outpatient clinic, then surgery was performed to repair the umbilical hernia. In this second operation, a 12-mm trocar was placed by the open method 3 cm below the inferior margin of the rib along the left midclavicular line (Palmer's point) (Fig. 3). In the first operation, the first port had been placed on the right side, and so, considering the possibility of adhesion, it was placed on the contralateral side for the second operation. Pneumoperitoneum was performed at 10 mmHg, and the abdominal cavity was observed with a 10-mm deflectable-tip videoscope (ENDOEYE FLEX™; Olympus). No adhesion was observed in the abdominal cavity. A 5-mm trocar was placed in the left lumbar region and surgery was performed with 2 ports. Observations from the abdominal cavity showed no signs of infection around the hernia, though an incisional scar was observed at the hernial orifice dilatated by incision which was measured to be 6 × 5 cm (Fig. 6a). We decided on IPOM as the operative method, and a VENTRALIGHT™ ST Mesh with ECHO 2™ Positioning System measuring 15.2 × 10.2 cm (C.R. Bard/Davol, Warwick, RI, USA) was used with sufficient margins for the hernial orifice. A supporting thread in the center of the mesh was hung from the center of the hernia in the umbilicus with EndoClose™, and the mesh was secured by the double-crown method using SorbaFix® (C.R. Bard/Davol) (Fig. 6b). The 12-mm trocar wound was sutured with 0 Monosyn® using EndoClose™ and all wounds were closed by dermal suturing with 4–0 Biosyn™. Lidocaine hydrochloride (1%) was infiltrated into the port sites at the end of surgery. The operating time was 54 min, and intraoperative hemorrhage was 1 mL. The postoperative course was uneventful, and the patient was discharged on postoperative day 4. No sign of infection or recurrence has been observed as of the time of writing, 8 months postoperatively.

## Discussion

Treatment of adult incarcerated umbilical hernia involves two procedures: early relief of the incarceration and closure of the hernial orifice. There appears to be no disagreement regarding techniques to relieve incarceration, but various approaches to closing the hernial orifice have been considered, such as laparoscopic or laparotomic methods, simple suture or mesh closure, and one- or two-stage operations, and no standard approach has yet been determined.

Detailed description or notation of each case of umbilical Littre's hernia were lacking in our systematic review of the literature [3] which searched PubMed using "Meckel's diverticulum, umbilical hernia" and "Littre's hernia, umbilical" for the period from 1955 to February 2020. Articles not written in English or referring to children or autopsy specimens were excluded, and 6 articles were identified [4–9] (Table 1). No reports describing laparoscopic treatment similar to this case were identified, with laparotomy performed in all cases.

All the hernia repairs were performed as one-stage procedures, and used simple suture closure in 3 cases, mesh in 2, and was not described in 1 case. The recurrence rate is reportedly significantly lower when using mesh as compared to simple suture [1, 2]. In addition, obese patients are at high risk of recurrence, and so the use of mesh appears particularly desirable [10]. We concluded that mesh repair was preferable in this case because of the severely obese patient.

In the two previous cases that used mesh, one reported a damaged Meckel's diverticulum that was adherent to the hernial orifice, and so ileal resection including the Meckel's diverticulum was performed. Onlay mesh was used for the hernia repair, and no postoperative mesh infection was observed although there was some superficial surgical site infection which required antibiotics [8]. In the other mesh repair case, no perforation was observed, but the diverticulum was dusky and discolored. Partial excision of the small intestine including the Meckel’s diverticulum was thus performed, and the hernia was repaired using interrupted sutures and reinforced with onlay mesh. No infection was identified.

Some consensus has been reached that the use of mesh is contraindicated in cases of severe intraperitoneal contamination with intestinal perforation. Mesh can reportedly be safely used in cases without intestinal resection following incarceration hernia [11]. However, the use of mesh for cases of intestinal resection with suspected infection remains contentious, and is generally considered contraindicated, because of the increased risk of implant infection [12]. On the other hand, some reports have found no difference in infection between repairs for incarcerated inguinal hernia with intestinal resection either with or without mesh [11, 13, 14], whilst another report has suggested that mesh should be used except in cases involving panperitonitis or extensive fecal contamination [15]. We believe that refraining from using mesh is advisable in any case where contamination is suspected, because treatment with antibiotics is difficult and mesh removal may be necessitated.

Using simple suture closure to avoid the risk of mesh infection during emergency surgery due to incarceration is questionable from the perspective of recurrence risk and curativeness, particular since mesh is ideal in terms of preventing recurrence. However, infection in this case had developed in the umbilical region due to an enterocutaneous fistula, and so we judged single-stage repair as carrying an overly high risk of mesh infection; the first operation was conducted solely to relieve the incarceration and resect the diverticulum. The infection was subsequently controlled and the IPOM repair was carried out in two stages so that the hernial repair could be safely performed. On the other hand, we think there are two problems for the two-stage surgery. One is the possibility of adhesion by first operation. To avoid this, we used Seprafilm, and we did not experienced adhesion at the second operation. The other is the risk of incarceration again. We need to explain the risk while waiting. And if incarcerate again, it is important to be able to respond promptly before intestinal necrosis. Careful follow-up is necessary because of this.

The IPOM repair was performed in this case using a two-port technique with one 12-mm and one 5-mm trocar. In laparoscopic ventral or incisional hernia repair, this two-port is less invasive than methods involving more than 2 ports, while the mean operative time is comparable to procedures with at least 3 [16]. Reducing the number of ports can also reduce the risk of port-site hernia, which frequently occurs in obese patients [17–19]. However, deploying and securing the mesh in the abdominal cavity using 2 ports requires refinement. Methods including suturing a supporting thread to lift the center of the mesh [20] and suturing the edge of the mesh at intervals of several centimeters [21, 22] have been used. The VENTRALIGHT™ ST Mesh with ECHO 2™ Positioning System used here employs a supporting thread at the center of the mesh, and its positioning system makes the mesh easier to handle. As a result, the two-port technique was performed quite easily.

In laparoscopic incisional hernia repair, IPOM plus repair involves the performance of IPOM repair after suture the abdominal wall defect. Comparing the two approaches, some investigations have suggested that IPOM plus repair reduces recurrence rate, the risk of seroma, and mesh bulge [23–26]. We acknowledge that IPOM plus repair may reduce the recurrence rate of umbilical hernia. However, in this case, the Meckel's diverticulum was not easily released from the hernia in the first operation, and the hernial orifice was hard and scarred from the incision. We thus judged suture closure to be too difficult and only performed the IPOM repair here.

We diagnosed an infected urachal remnant at first. There are no symptoms of bowel obstruction, so it was difficult to diagnose. In Littre’s hernia, Meckel’s diverticulum is the only incarcerated intestinal tract, and intestinal obstruction may not occur. Patients presented symptoms of bowel obstruction in Littre’s hernia was reported only 34.0% [3].

The possibility of patent omphalomesenteric duct can be considered as a differential diagnosis after surgery. This differential diagnosis is very difficult, especially if the process is long like this case. This patient is severely obese man with BMI 36.5 kg/m^2^. It was difficult to diagnose the incarcerated hernia without image diagnosis because the umbilical bulge could not be confirmed only by physical examination and palpation. It is difficult for a patient to be aware of umbilical bulge, even if he have had a history of spontaneous reduction. And there was no history of umbilical inflammation before this time. The preoperative course was close to 3 months, during which he was given antibiotics several time. It was considered that the incarcerated part of Meckel’s diverticulum did not perforate and reached the chronic inflammation phase, and to form an enterocutaneous fistula. Pathologically, Meckel’s diverticulum did not have necrotic changes. There were some fibrosis, its inflammatory changes was mild. At the first time operation, hernia sac thickening was observed macroscopically, which is probably due to chronic inflammation. Based on the above, we diagnosed as incarcerated umbilical hernia rather than patent omphalomesenteric duct.

## Conclusion

We encountered a very rare adult case of Littre's hernia was incarcerated within an umbilical hernia. A two-stage laparoscopic IPOM repair for incarcerated umbilical hernia appears useful in severely obese patients to prevent mesh infection and reduce recurrence rate.

**Table 1.** Six reports of Littre’s hernia

Author	Year	Age	Sex	Condition of Meckel's diverticulum	Diameter of hernial orifice	Surgical approach	Method of diverticulum excision	Method of hernia repair	Postoperative complications
Castleden [4]	1970	61	F	Damaged	N/A	Open	Wedge resection	Interrupted non-absorbable sutures	none
Tiu et al. [5]	2006	55	F	Dusky, discolored, non-perforated	N/A	Open	Partial resection of small bowel	Interrupted prolene sutures and reinforced with onlay prolene mesh	N/A
Sengul et al. [6]	2010	42	F	Discolored to grayish-black	3 cm	Open	Partial resection of small bowel	Herniorrhaphy	none
Kurnicki et al. [7]	2011	22	M	N/A	N/A	Open	N/A	Herniorrhaphy	none
Kibil et al. [8]	2012	35	M	Meckel's diverticulum adherent to hernia neck during dissection process, diverticulum was injured	3 cm	Open	Wedge resection	Onlay synthetic mesh prothesis(polypropylene mesh)	superficial surgical site infection
Cikman et al. [9]	2015	40	M	Fistula between Meckel's diverticulum and umbilicus	N/A	Open	Diverticulectomy	N/A	N/A

*N/A* not available, *M* male, *F* female, *Open* open laparotomy

## Data Availability

Not applicable.

## Abbreviations

IPOM: Intraperitoneal onlay mesh
CT: Computed tomography
